# Human neural correlates of emotional well-being (EWB): a preliminary systematic review and meta-analysis of MRI studies based on a recent consensus definition

**DOI:** 10.3389/fnhum.2025.1669164

**Published:** 2025-11-10

**Authors:** Jie Luo, Celinene M. Lay, Caroline G. Richter, Adam Turnbull, Fabio Richlan, Crystal L. Park, Fumiko Hoeft

**Affiliations:** 1 Department of Psychological Sciences, University of Connecticut, Storrs, CT, United States; 2 Yale School of Medicine, Yale University, New Haven, CT, United States; 3 Department of Psychology, The University of Alabama at Birmingham, Birmingham, AL, United States; 4 Department of Psychiatry and Behavioral Sciences, Stanford University, Stanford, CA, United States; 5 Department of Psychology, Centre for Cognitive Neuroscience, Paris Lodron Universitat Salzburg, Salzburg, Austria; 6 Department of Neuropsychiatry, Keio Gijuku Daigaku Igakubu Daigakuin Igaku Kenkyuka Kaibogaku, Shinjuku, Japan

**Keywords:** emotional well-being (EWB), systematic review, meta-analysis, MRI, fMRI, incentive and rewards processing, social cognition, interoceptive awareness

## Abstract

**Introduction:**

Emotional well-being (EWB) is a multifaceted construct essential for human health, conceptualized as an umbrella term for related psychometric concepts such as psychological well-being (PWB), positive mental health, health-related quality of life, thriving, and subjective well-being (SWB). However, varying definitions have prompted calls for a consensus definition. Understanding the neural mechanisms of EWB is crucial for health and intervention efforts, yet findings remain inconsistent in both empirical studies and systematic reviews. The inconsistencies in prior systematic reviews may arise from diverse definitions, an emphasis on task-independent over task-dependent modalities, and biases introduced when statistical analyses are lacking.

**Methods:**

To address these gaps, this study presents the first preliminary systematic review and meta-analysis of the neural correlates of EWB using a consensus definition developed in 2023 by NIH EWB Research Network, which includes five domains: goal pursuit, life satisfaction, positive affect, quality of life, and sense of meaning. Importantly, we used a hypothesis-driven approach to separately examine task-dependent (task-based fMRI; *n* = 14) and task-independent modalities (resting-state fMRI and structural MRI; *n* = 7 each), clarifying their distinct and overlapping neural contributions of EWB.

**Results:**

The left pallidum as a key region associated with task-dependent modality, likely reflecting incentive and rewards processing, while task-independent findings implicate the right superior temporal gyrus (STG) and insula, suggesting roles in social cognition and interoceptive awareness. Across both modalities, frontoparietal regions emerge as shared substrates likely contributing to cognitive control processes central to EWB.

**Conclusion:**

Despite limited sample sizes, this review provides a preliminary neural framework of EWB, highlighting distinct and shared contributions across modalities and lay an empirical foundation for future large-scale investigations.

**Systematic review registration:**

https://osf.io/ymtb8/overview

## 1 Introduction

Emotional well-being (EWB) encompasses various psychological aspects, such as life satisfaction, life purpose, and positive emotions, reflecting how positively individuals feel about themselves and life overall (Park et al., 2023). Research consistently shows that higher EWB is linked to positive health outcomes, making it a critical focus in public health efforts. A meta-analysis of over 136,000 individuals revealed a 17% reduction in all-cause mortality associated with a strong sense of purpose (Cohen et al., 2016). A recent meta-analysis, which included over 214,270 individuals, found that a stronger sense of meaning and purpose in life, aspects of EWB, have a robust association with a lower risk of developing dementia (Sutin et al., 2023). These findings underscore EWB’s critical role in public health, as the goals of Healthy People 2030 and the NCCIH Strategic Plan 2021–2025 emphasize enhancing well-being to improve health and quality of life (Office of Disease Prevention and Health Promotion [ODPHP], 2021; Office of National Center for Complementary and Integrative Health [NCCIH], 2023; Koh et al., 2021).

The significance of EWB for human health is widely acknowledged, leading to various diverse definitions and measurements over the years. In 2018, Feller and colleagues conceptualized EWB as “an umbrella term for several related psychometrically defined concepts, including psychological well-being, positive mental health, health-related quality of life, thriving, and subjective well-being” (Feller et al., 2018). Additionally, alternative conceptualizations of EWB have introduced terms like well-being (WB) and subjective well-being (SWB). Diener et al. (2018) defined SWB as encompassing overall life appraisals and emotional experiences. The lack of consensus on EWB definitions and measures has led to confusion, prompting calls for a unified approach (VanderWeele et al., 2020).

To address this need, a working group supported by the National Institutes of Health (NIH) and comprised of six networks across the U.S. proposed a consensus definition of EWB (Park et al., 2023), covers the following domains: goal pursuit, life satisfaction, positive affect, quality of life, and sense of meaning. The working definition of EWB is: EWB is a multi-dimensional composite that encompasses how positive an individual feels generally and about life overall. It has both experiential features such as the emotional quality of everyday experiences and reflective features such as judgments about: life satisfaction, sense of meaning, and ability to pursue goals that can include and extend beyond the self. These features occur in the context of culture, life circumstances, resources, and life course. To facilitate research, the group also developed the “EWB Subjective Measurement Repository” (M3EWB Network, 2023), offering a range of tools to accommodate varied study contexts that list a range of instruments to meet diverse research needs. Despite these advances, challenges persist in studying EWB; as Park et al. (2023) highlight, there remains a need to explore the structure, predictors, and outcomes underlying EWB through diverse methodologies, such as neuroimaging. However, neuroimaging studies face additional complexities; for example, diverse EWB constructs, and measurement tools have contributed to varied findings on the neural correlates of EWB. The resulting heterogeneity complicates systematic review and meta-analytic efforts, hindering a comprehensive understanding of EWB’s neurobiological underpinnings. Overcoming these obstacles requires harmonizing EWB definitions and measures to systematically explore their neural correlates.

As Campos and Sanchez Hernandez (2023) noted, EWB research has largely relied on subjective measures, such as self-report questionnaires, which are cost-effective but vulnerable to biases from cognitive distortions and emotional states (Jahedi and Méndez, 2014). These limitations underscore the need for a multidisciplinary approach that integrates subjective and objective measures. Combining neuroscience, psychology, and related fields offers a more comprehensive way to study EWB. Neuroimaging, though limited in establishing causality (Weber and Thompson-Schill, 2010), provides objective biomarkers less affected by self-report biases and helps uncover neural processes underlying EWB. For example, sustained engagement of the brain’s rewards circuitry has been linked to higher EWB (Heller et al., 2013), illustrating how such methods can reveal mechanisms beyond subjective reports. This is emphasized by the NCCIH Strategic Plan 2021–2025, which calls for investigating the neural bases of EWB using tools like neuroimaging and computational modeling to optimize interventions. Magnetic resonance imaging (MRI), a widely used technique, enables examination of both brain structure and function. Functional MRI (fMRI) tracks task-related activity via blood flow changes (Logothetis, 2008), while structural MRI captures anatomical features. Task-dependent (task-fMRI) approaches identify brain regions activated by specific tasks, whereas task-independent methods (resting-state fMRI, structural MRI) explore intrinsic connectivity and organization (Biswal et al., 1995). Despite these advances, our understanding of EWB’s neural mechanisms remains limited, with empirical studies reporting inconsistent findings. Some have linked EWB to regions like the insula, associated with self-awareness and emotional control (Lewis et al., 2014; Kong et al., 2015a), while others implicate broader networks, including the salience and default mode networks (King, 2019; Kringelbach and Berridge, 2009).

Despite growing interest in the neuroscience of EWB, findings across studies remain inconsistent. Several key methodological and conceptual limitations have likely contributed to this variability. First, previous research has lacked a unified definition of EWB, resulting in heterogeneity across studies in terms of included constructs and measures. This lack of conceptual consensus has hindered efforts to consolidate findings and identify reliable neural correlates.

Second, many prior systematic reviews (King, 2019; de Vries et al., 2023; Richter et al., 2024) have focused exclusively on task-independent neuroimaging modalities (e.g., resting-state fMRI and structural MRI), without focusing on task-dependent modalities (e.g., task-based fMRI) that can more directly probe functional processes related to EWB, such as emotion regulation and rewards responsiveness (Park et al., 2023). For example, a recent review examined the link between task-independent brain features and well-being, concluding that there are no consistent neural correlates of EWB (de Vries et al., 2023). However, task-based fMRI studies have used some paradigms (e.g., emotion processing) to correlate task-related brain activity with EWB self-report scores, providing insights into the neural correlates of EWB within specific contexts. Integrating task-dependent and task-independent approaches offers a dual advantage: it highlights the functional relevance of neural connections during specific processes while also providing a broader understanding of brain organization (Stevens, 2016). Therefore, to achieve a comprehensive framework for understanding the neural correlates of EWB, it is necessary to adopt a broader and more integrative scope.

Third, many of the existing systematic reviews on the neural correlates of EWB have provided valuable insights across studies (King, 2019; de Vries et al., 2023; Richter et al., 2024). Reflecting the early stage of this research area, many of these seminal studies were explored in nature and not guided by formal hypotheses. While exploratory studies have been instrumental in identifying candidate brain regions and circuits, the field now requires more theory-informed approaches to advance reproducible and testable models. As Platt (1964) argued, scientific progress depends on strong inference and clear hypotheses, especially in complex domains such as affective neuroscience.

Fourth, although systematic reviews have synthesized qualitative patterns, few have employed quantitative meta-analyses, particularly in the context of EWB (King, 2019; de Vries et al., 2023; Richter et al., 2024). Without statistical integration, such reviews may inadvertently introduce subjective bias and lack the power to detect convergent neural correlates. Quantitative meta-analyses, such as activation likelihood estimation (ALE), offer a robust framework for pooling data across heterogeneous studies and identifying consistent activation patterns across tasks and modalities (Eickhoff et al., 2009), even at preliminary stages when sample sizes remain modest.

Finally, to our knowledge, no previous meta-analysis of neuroimaging studies has examined the neural correlates of EWB which applied the NIH-supported consensus definition (Park et al., 2023), that encompasses goal pursuit, life satisfaction, positive affect, quality of life, and sense of meaning. The recent availability of this definition, along with the EWB Subjective Measurement Repository (M3EWB Network, 2023), provides an unprecedented opportunity to reframe and systematically evaluate the neuroimaging literature using a standardized framework. The current study addresses these gaps by:

1. Utilizing the consensus definition of EWB developed by the NIH EWB Research Network (Park et al., 2023) to guide study selection and classification.
2. Using a hypothesis-driven approach to conduct a systematic review and preliminary quantitative meta-analysis of neuroimaging studies that examine the neural correlates of EWB, including both task-independent and task-dependent modalities. Based on prior work (Alexander et al., 2021), we hypothesized that in task-dependent modality, regions linked to the dopaminergic system—such as the amygdala, striatum, and pallidum—would be associated with EWB, given their roles in positive reinforcement and rewards regulation, which are essential for sustaining emotional well-being (Santos et al., 2016; Schultz, 2016; Berridge and Kringelbach, 2015; Haber and Knutson, 2010). Positive reinforcement enhances behaviors tied to pleasure and satisfaction, while rewards regulation supports emotional resilience. For task-independent modality, we expected consistent associations with regions such as the temporal-parietal junction (TPJ) and insula, known to contribute to interoceptive awareness and social cognition. Interoceptive awareness helps regulate emotions by interpreting internal bodily states, while social cognition—including empathy and theory of mind—strengthens social relationships that underpin emotional stability and overall EWB (Koban et al., 2021; Cacioppo and Hawkley, 2009).
3. Comparing findings across modalities to delineate both distinct and shared neural substrates associated with EWB.

This integrative and hypothesis-driven approach provides an essential foundation for advancing the neuroscience of EWB and informing future intervention development.

## 2 Methods

### 2.1 Literature searching, screening, coding, and quality assessment

This study was built upon the scoping review by Richter et al. (2024) and followed PRISMA 2020 guidelines (Page et al., 2021) for the literature search. We screened articles from five electronic databases: PubMed, PsycINFO, Web of Science, ERIC (EBSCO), and Embase, with the most recent search conducted on 14 March 2024. Our search did not include specific publication dates to capture the maximum number of relevant articles. As Table 1 shows, we used keywords combining terms related to EWB and neuroimaging modalities, including articles with at least one term in the following three groups: Group A (EWB components), Group B (brain imaging modalities), and Group C (neuro-related terms). The search process involved title, abstract, and full-text screening using Covidence systematic review software (Veritas Health Innovation, 2025). We included many neuroimaging modalities in the search to ensure a comprehensive search, but retained only MRI studies based on current study objectives. This also maintains consistency with the methodology of Richter et al. (2024), facilitating comparability across reviews and enhancing the transparency, reproducibility, and reliability of our search process. Covidence is an online collaboration tool specifically designed for conducting systematic reviews. The platform enables multiple users to efficiently manage literature screening, data extraction, and risk of bias assessment, making it particularly suitable for systematic reviews and meta-analyses that follow PRISMA guidelines.

**TABLE 1:** Search terms utilized.

Search terms utilized	
Group A	Emotional well-being OR emotional wellbeing OR psychological well-being OR psychological wellbeing OR subjective well-being OR subjective wellbeing OR life satisfaction OR happiness OR happy OR positive emotion* OR flourish* OR Eudaimoni* OR evaluative well-being OR evaluative wellbeing OR hedonic well-being OR hedonic wellbeing OR experiential well-being OR experiential wellbeing OR spiritual well-being OR spiritual wellbeing OR positive affect OR meaning in life
Group B	“Magnetic resonance imag*” OR “functional MRI” OR electroencephalogra* OR “event related*” OR event-related* OR “magnetic resonance spectroscop*” OR “positron emission” OR “single-photon emission” OR magnetoencephalogra* OR “Transcranial magnetic stimulation” OR “Transcranial direct current stimulation” OR “diffusion weighted” OR “diffusion-weighted” OR “diffusion tensor” OR “diffusion-tensor” OR “diffusion MRI” OR “diffusion imaging” OR MRI OR fMRI OR EEG OR ERP OR MRS OR PET OR SPECT OR MEG OR TMS OR tDCS OR DWI OR DTI
Group C	brain* OR neur*

Group A, B, and C were combined with AND.

The inclusion criteria for this study were (1) At least one MRI modality (structural MRI, resting-state fMRI, or task-based fMRI); (2) At least one measure of EWB (or its components); (3) Articles published in peer-reviewed journals; (4) Studies published in English; (5) Studies reported a correlation coefficient between brain metrics (e.g., gray matter volume, brain activation, etc.) and EWB scores from the EWB subjective measurements repository^1^ ; (6) Aimed to include a broad range of populations as much as possible to explore EWB across different demographics and conditions; and (7) Whole-brain analysis used approaches in the initial step. Only studies initially employing whole-brain analysis were included to avoid bias (Müller et al., 2018). All task-based fMRI studies included in this review began by analyzing whole-brain activity to identify significant clusters, then examining the regions significantly correlated with EWB, reducing bias due to researchers’ choice of specific regions. While this analysis may still bias the results toward regions that are active at the group level during the performance of the specific task used in each study, we feel that this is a reasonable compromise given the challenges of accessing un-thresholded task-fMRI maps from previous studies. However, we acknowledge the potential for Type II error: some important regions may be missing from our results, which should be considered when interpreting the findings.

**FIGURE 1:**
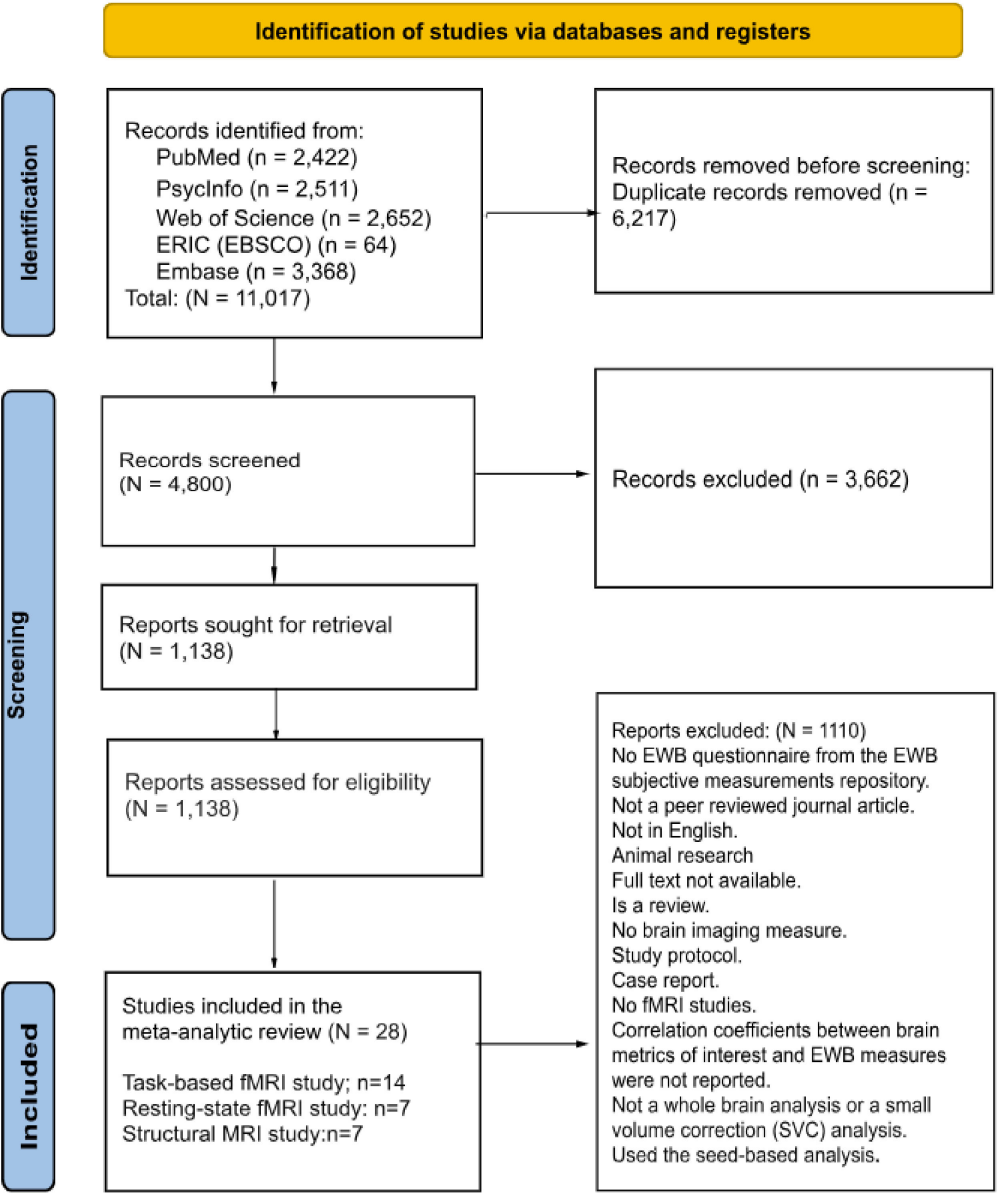
PRISMA flowchart of article selection processing.

The exclusion criteria included: (1) Book chapters, reviews, case studies, qualitative studies, meta-analyses, and systematic reviews; (2) Unrelated articles, duplicates, unavailable full texts, or abstract-only papers; (3) Articles published only in Google Scholar, dissertations, theses, conference papers; (4) Animal research; and (5) Seed-based studies. The exclusion of seed-based studies was necessary because they present a challenge due to the diverse selection of seed regions. Researchers often choose these seed regions based on their hypotheses or previous findings. This variability in seed selection makes it difficult to establish consistent and comparable criteria for inclusion in a study. The PRISMA flowchart (Figure 1) details the literature selection process.

After screening the literature, we assessed the quality of the included studies using Covidence systematic review software, recognizing that evaluating reliability is essential (Dreier, 2013). We assessed each article based on the following criteria: (1) Clarity of research hypotheses and design; (2) Power analysis for its sample size; (3) Standardized MRI data processing; (4) Clarity of results reporting; and (5) Availability of data or code. This assessment aimed to gauge the reliability of each study but did not exclude articles. However, studies that lacked the necessary information for the activation likelihood estimation (ALE) analysis (e.g., missing coordinates) were excluded. For specific quality assessment questions, see Supplementary Table 1. To ensure interrater reliability (Belur et al., 2021), our literature coding team, comprising three experienced researchers (JL, CL, GL), utilized Covidence software for structured coding and data extraction. We manually extracted peak coordinates of brain regions significantly correlated with EWB scores using the Montreal Neurological Institute (MNI) template for standardization. Coordinates provided in other templates, such as Talairach space, were converted to MNI using appropriate software^2^. We also manually coded additional variables, including participants’ mean age, study sample size, and effect sizes of reported correlations, to enable comprehensive analysis in the study. The interrater reliability, as measured by the intraclass correlation coefficient (ICC), ranged from 0.9 to 1.0 across all extracted variables, indicating excellent interrater reliability (Koo and Li, 2016) in the data coding process from the articles included in this study. The research protocol of this study was pre-registered (Luo et al., 2025) in Open Science Framework^3^.

### 2.2 Systematic review procedure

Following a literature search, screening, coding, and quality assessment, this study conducts a systematic review, separately examining task-dependent fMRI and task-independent MRI studies to identify the neural correlates of EWB. For each eligible study, we systematically extract and synthesize key variables, including brain metrics used, EWB measurement tools, reported significantly correlated brain regions, study population characteristics, and task fMRI paradigms for task-dependent studies. To maintain consistency, our study only included activation metrics for task-based fMRI studies, gray matter volume for structural MRI studies, and metrics such as regional homogeneity (ReHo) and fractional amplitude of low-frequency fluctuation (fALFF) for resting-state fMRI studies, which quantify spontaneous neural activity without any task (Zang et al., 2004; Zou et al., 2008). The synthesized findings aim to provide a clearer understanding of the consistently reported significant brain regions with EWB across different studies, helping to identify common patterns and methodological gaps in the literature.

### 2.3 Preliminary meta-analysis procedure

#### 2.3.1 Activation likelihood estimation (ALE)

To identify consistent activations, spontaneous activities, or reported anatomic metrics across previous studies that correlate with EWB, we conducted an ALE analysis (Eickhoff et al., 2009) using Ginger ALE software (version 3.0.2^4^). ALE performs a coordinate-based meta-analysis to determine where brain results converge at an above-chance level. We set a cluster-level familywise error (FWE) threshold of *P* < 0.05 and a voxel-level of *P* < 0.001 with 1,000 permutations, a standard approach in meta-analytic studies. In synthesizing data for our meta-analysis, we focused on significant peak coordinates extracted from studies, which typically come from thresholded maps. While we acknowledge that thresholded maps may introduce bias, un-thresholded maps are often challenging to obtain. For the ALE results, we reported MNI peak coordinates, cluster volumes, ALE scores, Z scores, *P*-values, and cluster breakdowns.

Eickhoff et al. (2016) recommend including between 8 (voxel-level FWE) and 17 (cluster-level FWE) individual experiments in an ALE meta-analysis to reduce bias and ensure adequate statistical power. However, this number depends on the expected effect size; smaller sample sizes may suffice for strong effects, while small to medium effects require caution if fewer experiments are included (Müller et al., 2018). Due to the lack of task paradigms that directly measure EWB, the current study’s analysis, including literature, relies on existing task paradigms within the task-dependent modality to identify the neural correlates of EWB.

#### 2.3.2 Jackknife analyses and publication bias

To minimize the impact of individual studies and reduce the risk of false positives in our meta-analyses, we used ALE (version 3.0.2) to do the leave-one-out (jackknife) analysis (Müller et al., 2018). This method systematically excludes one experiment at a time and reruns the analysis to check if any meta-analytic clusters depend heavily on a single study, thereby enhancing the reliability of our findings. Additionally, we assessed publication bias using Egger’s test (Egger et al., 1997) and funnel plots to determine if biased reporting affected the results (Müller et al., 2018; Lin and Chu, 2018). Publication bias in meta-analysis refers to the phenomenon where studies with positive or significant results are more likely to be published. In contrast, those with negative or non-significant results are less likely to be published or may remain unpublished (Van Aert et al., 2019). This can lead to a skewed representation of the evidence when conducting a meta-analysis. A significance level of *p* < 0.05 in Egger’s test indicated significant publication bias (Egger et al., 1997). Further details are provided in Supplementary Table 1.

#### 2.3.3 Comparative analysis of task-dependent and task-independent modalities

After analyzing neural correlation patterns to obtain meta-maps, we computed conjunction and contrast maps, which is the approach used in other studies (Martinez-Lincoln et al., 2023), to identify shared and distinct features between task-dependent (task-based fMRI) and task-independent (resting-state fMRI) modalities; task-dependent (task-based fMRI) and task-independent (structural MRI) modalities. Statistically, we first created a pooled map of foci, adding maps of both modalities. The foci were then randomly divided into two equal-sized groups 1,000 times, comparing the resulting difference map to an empirical null distribution. We set a *P*-value threshold of 0.01 and a cluster size of 500 mm^3^, using 1,000 permutations in Ginger ALE software (Eickhoff et al., 2009). In cases where comparative analysis in Ginger ALE cannot be conducted due to an insufficient sample size, a descriptive approach will also be applied across all comparisons to illustrate similarities and differences between modalities.

#### 2.3.4 Neurosynth cognitive decoder

To further explore our findings and infer underlying processes contributing to EWB, we used Neurosynth, an automated meta-analysis platform, to decode terms associated with EWB from our ALE analysis results (Yarkoni et al., 2011)^5^. Neurosynth analyzes over 14,300 functional neuroimaging studies reporting data from over 150,000 brain regions and includes a database of over 1,300 terms extracted from studies that can be associated with the brain coordinates of the corresponding studies. In that case, in its extensive database, this platform can support the “decoding” of a wide range of cognitive states by brain activity and peak coordinates from all neuroimaging studies. We uploaded our ALE maps for both task-dependent and task-independent modalities to the NeuroVault repository^6^, allowing us to compare our data with studies in the Neurosynth database (Gorgolewski et al., 2015). Using reverse inference, we identified 1,307 terms correlated with EWB meta-maps, focusing on the top 50 relevant terms (retrieved date: October 20*^th^*, 2024). After excluding brain regions and methodological terms, we compiled a list of neurocognitive terms along with their corresponding correlation scores.

## 3 Results

### 3.1 Task-based fMRI studies results from task-dependent modality

**FIGURE 2:**
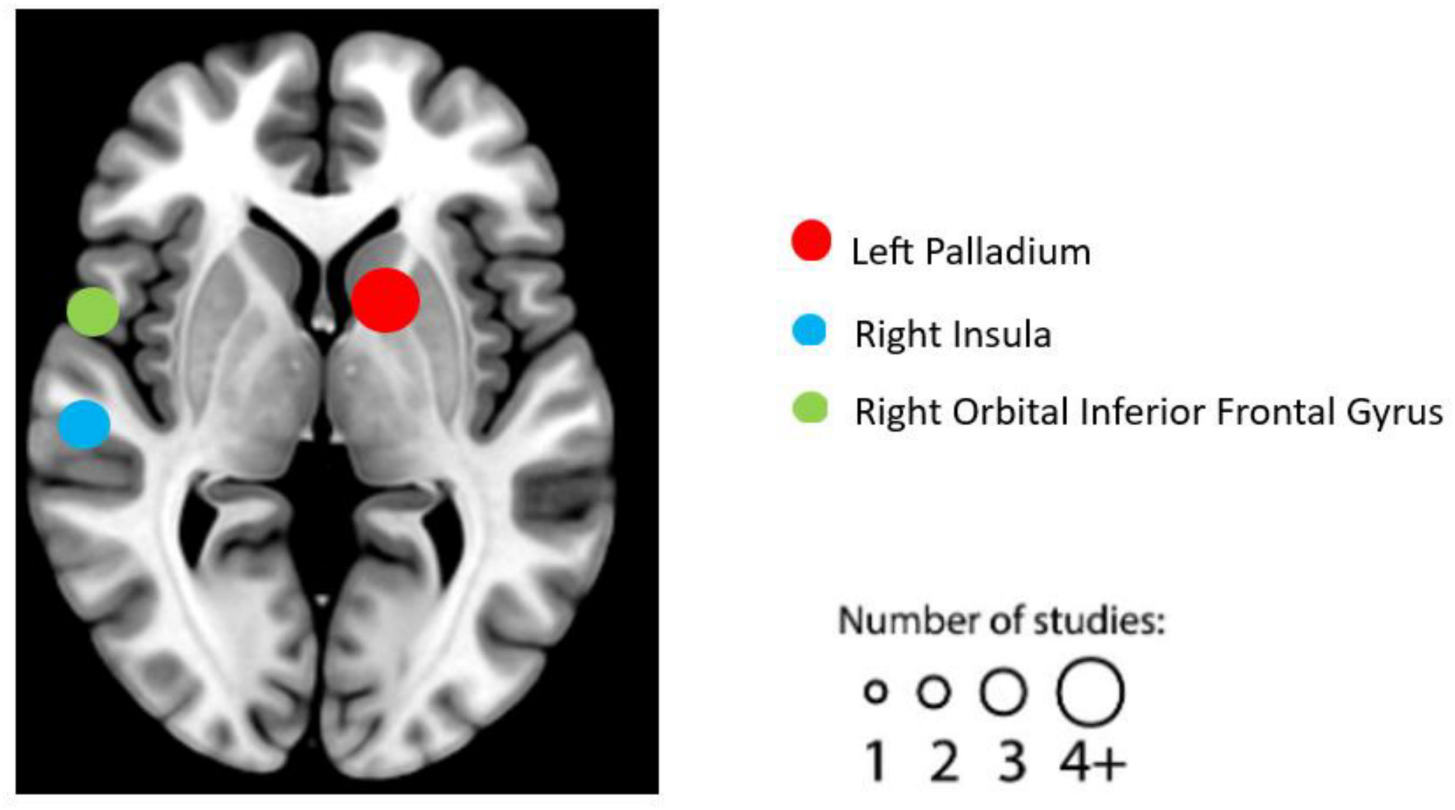
Graphical illustration of frequently reported task functional magnetic resonance imaging (fMRI) findings correlated with emotional well-being (EWB). Circles are sized according to the number of experiments reporting findings in the given location.

Following a comprehensive literature review and subsequent coding process, we identified 16 individual experiments eligible for the task-based fMRI studies. See Table 2 for details on the design, characteristics, and reported results of each experiment. We summarized the reported brain regions from the included experiments based on their frequency to identify patterns. Based on the characteristics of each included experiment, the most frequently reported brain regions correlated with EWB are the left palladium (five individual experiments reported), followed by right orbital inferior frontal gyrus (three individual experiments reported) and right insula (two individual experiments reported) (see Figure 2).

**TABLE 2:** The design characteristics and results of the task-based functional magnetic resonance imaging (fMRI) studies in task-dependent modality to emotional well-being (EWB).

References	Brain metrics	*N*	Age mean (SD)	Population description in the study	Task fMRI paradigm	EWB measurement	Reported significant correlated regions
Heller et al., 2013	Activation	64	(-)	Adults	International affective picture recognition task (Lang et al., 1998)	Psychological well-being, Positive and Negative Affect Scale -Gen	R_Caudate
							R_Middle Frontal
							R_Caudate
Shi et al., 2016	Activation	50	20.88 (1.51)	University students	Self-evaluation task: personality trait adjective pool	General life satisfaction scale	L_Caudate
							L_Pallidum
							R_Dorsal Striatum
							R_Middle Superior Frontal Gyrus
							L_ Inferior Parietal Lobe
Zhang et al., 2015	Activation	25	18.61 (-)	University students	Chinese affective picture system recognition task	Positive and Negative Affect Scale	R_Orbital Inferior Frontal Gyrus
							R_Putamen
							L_Orbital Inferior Frontal Gyrus
							L_Amygdala
Van Reekum et al., 2007	Activation	25	63.50 (-)	Adults	International affective picture recognition task (Lang et al., 1998)	Ryffs Scales of Psychological Well-being	L_Anterior Cingulum Gyrus
Sanchez et al., 2015	Activation	22	26.32 (4.52)	Adults	Discrimination/ Judgment task	Positive and Negative Affect Scale	L_Amygdala
							R_Hippocampus
Park et al., 2017	Activation	24	(-)	Adults- experimental group	Decision-making task	Subjective happiness scale	L_Pallidum
Park et al., 2017	Activation	24	(-)	Adults- control group	Decision-making task	Subjective happiness scale	L_Pallidum
Morelli et al., 2018	Activation	46	19.33 (1.23)	Adults	Monetary incentive delay task	Positive and Negative Affect Scale, Satisfaction with Life scale	L_Putamen
Memarian et al., 2017	Activation	111	21.00 (3.30)	University students	Affect labeling task	Satisfaction with Life scale	L_Superior Motor Area
							R_Medial Superior Frontal Gyrus
							L_Paracingulate gyrus
							L_Inferior Frontal Gyrus
							R_Medial Superior Frontal Gyrus
							L_ Middle Frontal Gyrus
							R_Inferior Frontal Gyrus
							R_Superior Frontal Gyrus
							R_Orbital Inferior Frontal Gyrus
Matsunaga et al., 2016	Activation	106	21.40 (-)	University students	Life event imagination task	Subjective happiness scale	Rostral anterior cingulate
							cortex
Martin-Soelch et al., 2021	Activation	16	25.19 (4.79)	Health adults	Monetary rewards: fribourg rewards task	Positive and Negative Affect Scale	L_Pallidum
Martin-Soelch et al., 2021	Activation	16	24.31 (4.08)	Family history of depression adults	Monetary rewards: fribourg rewards task	Positive and Negative Affect Scale	L_Pallidum
Killgore and Yurgelun-Todd, 2007	Activation	13	23.50 (2.10)	Adults	Stimulation Tasks: viewing images of high-calorie and low-calorie foods	Positive and Negative Affect Scale	R_Lingual Gyrus
Fonzo et al., 2017	Activation	36	36.73 (-)	PTSD	Emotion reactivity task (Etkin et al., 2004)	WHO Quality of Life Brief scale	L_Superior Frontal Gyrus
Mathiak et al., 2013	Activation	13	(-)	Adults	Playing a violent video game	Positive and Negative Affect Scale	R_Orbital Inferior Frontal Gyrus
							L_Superior Temporal Pole
							Ventra Medial prefrontal cortex
							R_Precuneus
							R_Hippocampus
Murray et al., 2023	Activation	82	15.83 (1.89)	Adolescent with anhedonia	Monetary rewards task	Positive and Negative Affect Scale	R_Insula
							R_Insula
							R_Insula

“-” denotes information not reported in the corresponding articles. In the “Reported significant correlated regions” column, “L” refers to the left hemisphere, and “R” refers to the right hemisphere.

**FIGURE 3:**
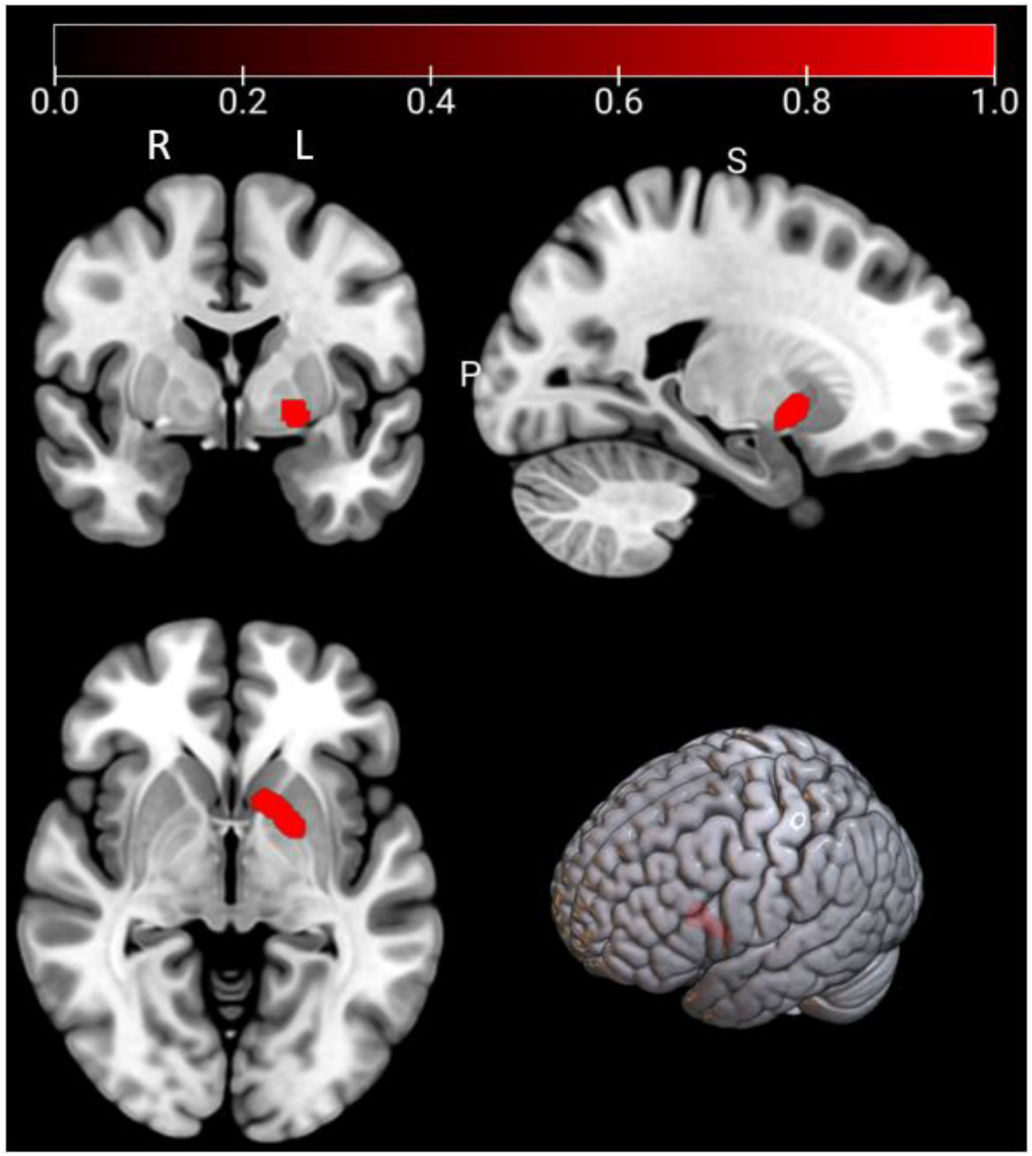
Emotional well-being (EWB) meta-maps (marked with red) of task-based functional magnetic resonance imaging (fMRI) studies (the task-dependent modality). “L” refers to the left hemisphere, and “R” refers to the right hemisphere.

As shown in Figure 3 and Table 3, according to the ALE result, a significant cluster of task-based fMRI studies was observed in the left pallidum in this ALE meta-analysis. Jackknife analyses have also further validated these findings, indicating the robustness and high replicability in the observed cluster: left pallidum (15 out of 16 replicabilities). See Supplementary Table 3 for more details.

**TABLE 3:** Emotional well-being (EWB) meta-maps in task-based functional magnetic resonance imaging (fMRI) studies from task-dependent modality.

Clusters	Regions	L/R	MNI coordinate			ALE score	*P*	Z	Voxels (mm^3^)	Jackknife sensitivity analysis
1	Pallidum	L	−16	8	2	0.0216	6.22E-09	5.6935	1992	15/16
	Pallidum	L	−20	2	−4	0.0177	2.29E-07	5.0434	–	–

In the “Reported significant correlated regions” column, “L” refers to the left hemisphere, and “R” refers to the right hemisphere.

### 3.2 Resting-state fMRI studies results from task-independent modality

**FIGURE 4:**
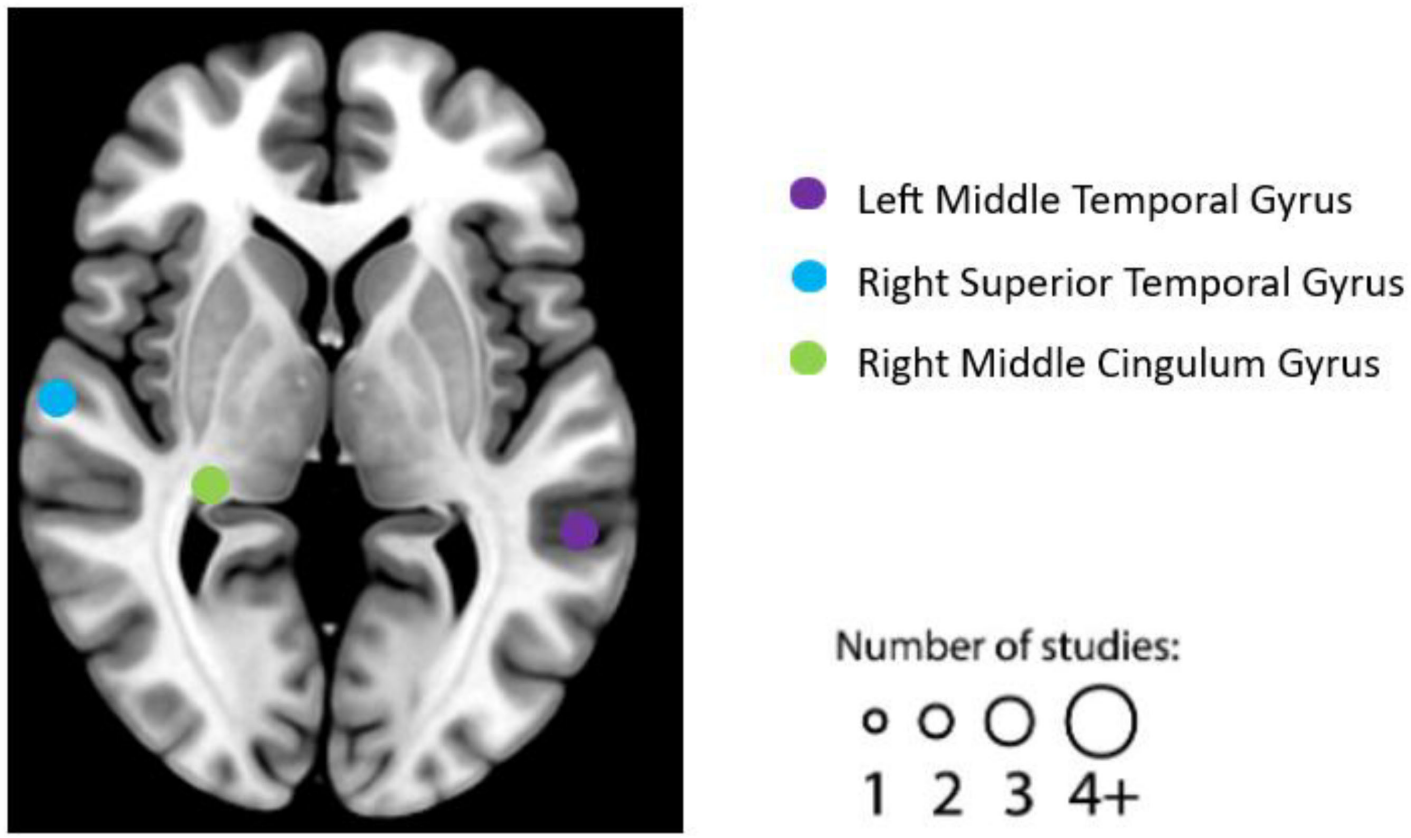
Graphical illustration of frequently reported resting functional magnetic resonance imaging (fMRI) findings correlated with emotional well-being (EWB). Circles are sized according to the number of experiments reporting findings in the given location. “L” refers to the left hemisphere, and “R” refers to the right hemisphere.

After literature screening and coding, seven individual experiments were identified as qualified for the resting-state fMRI studies. See Table 4 for details on the design, characteristics, and reported results of each experiment. We summarized the reported brain regions from the included experiments based on their frequency to identify patterns. Based on the characteristics of each included experiment, the most frequently reported brain regions correlated with EWB are the right STG (two individual experiments reported), left middle temporal gyrus (two individual experiments reported), right middle cingulum gyrus (two individual experiments reported) (see Figure 4).

**TABLE 4:** The design characteristics and results of the task-independent modality [resting-state functional magnetic resonance imaging (fMRI) studies] to emotional well-being (EWB).

References	Brain metrics	*N*	Age mean (SD)	Population	EWB measurement	Reported significant correlated regions
Kong et al., 2015a	ReHo	276	21.57 (1.01)	Adults	Satisfaction with Life scale	R_Middle Cingulum Gyrus
Sato et al., 2019	fALFF	51	22.50 (4.50)	Adults	Subjective happiness scale	R_Cuneus
						L_Precuneus
						R. Superior parietal lobule
						R_Precuneus
Kong et al., 2018	fALFF	95	(-)	Healthy individuals	Positive and Negative Affect Scale, Satisfaction with Life scale	L_Orbital Frontal Gyrus
						L_Orbital Frontal Gyrus
Kong et al., 2016	ReHo	290	21.56 (1.01)	Healthy university students	Positive and Negative Affect Scale, Ryffs Scales of Psychological Well-being-18	R_Precentral Gyrus L_Superior Temporal Pole
Kong et al., 2015b	fALFF	286	21.55 (1.01)	Healthy university students	42-item Scales of Psychological Well-being	R_Superior Temporal Pole
						L_Thalamus
Kong et al., 2015c	fALFF	294	21.56 (1.00)	Healthy university students	Satisfaction with Life Scale, Positive and Negative Affect Scale	L_Postcentral Gyrus
						R_Superior Temporal Gyrus
						L_Superior Temporal Gyrus
						R_Middle Cingulum Gyrus
						R_Lingual Gyrus
						R_Thalamus
						L_Middle Temporal Gyrus
						L_Superior Frontal Gyrus
						R_Superior Frontal Gyrus
						L_Inferior Temporal Gyrus
						R_Rectus Gyrus
						R_ Amygdala
Li et al., 2022	ReHo	60	37.80 (13.10)	Healthy individuals	14-item version of MHC-SF	R_Superior Temporal Gyrus
						L_ Superior Parietal Lobule
						L_Middle Temporal Gyrus

“-” denotes information not reported in the corresponding articles. “ReHo” stands for “regional homogeneity”; and “fALFF” stands for “fractional amplitude of low-frequency fluctuation.”

**FIGURE 5:**
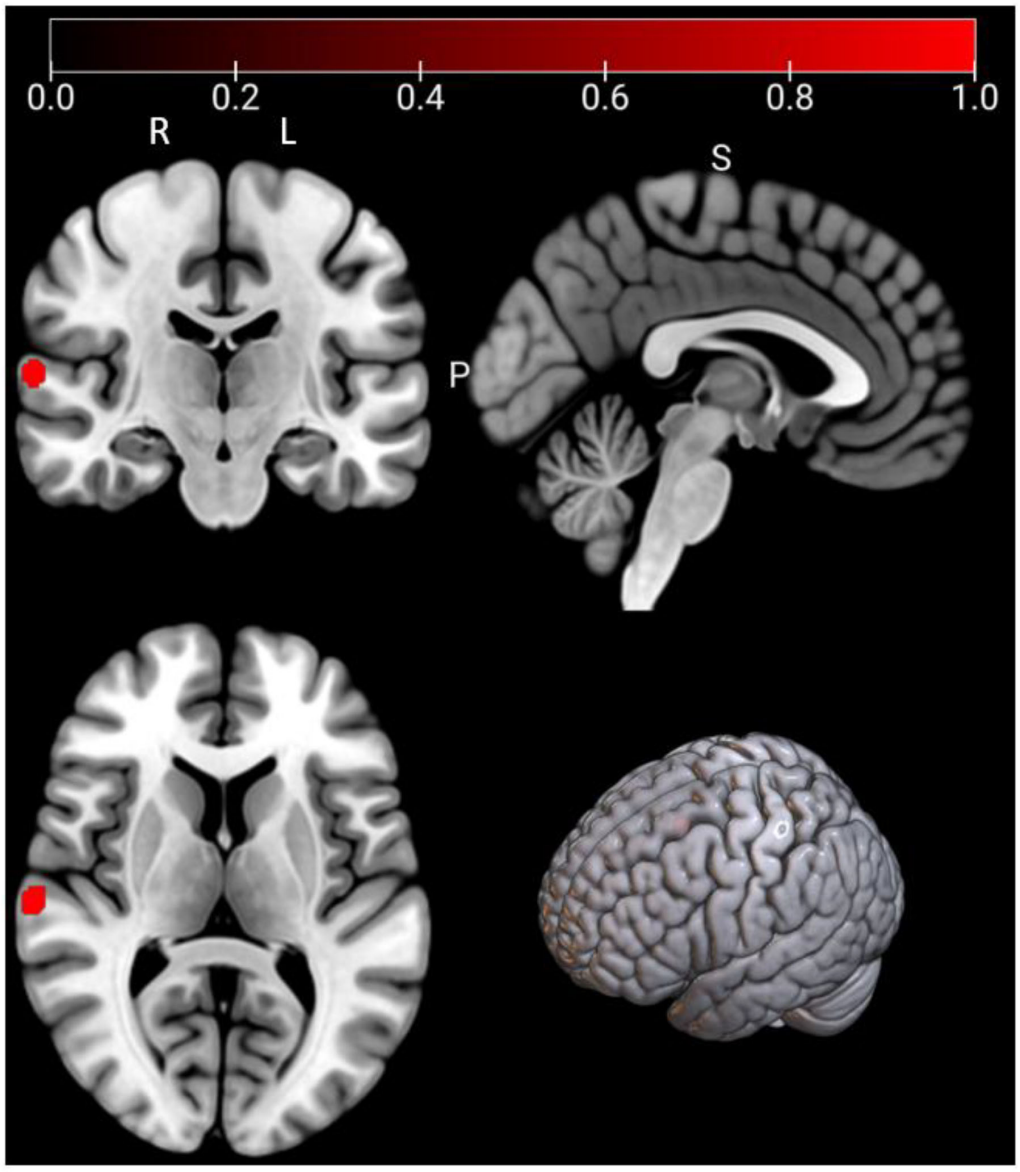
Emotional well-being (EWB) meta-maps (marked with red) of resting-state functional magnetic resonance imaging (fMRI) studies (the task-independent modality). “L” refers to the left hemisphere, and “R” refers to the right hemisphere.

As Figure 5 and Table 5, a significant cluster was observed in the ALE meta-analysis results of resting-state fMRI studies in the right STG. This finding was also further validated by jackknife analyses, indicating robustness and high replicability in both observed clusters of right STG (6 out of 7 replicabilities); see Supplementary Table 4 for more details.

**TABLE 5:** Emotional well-being (EWB) meta-maps in resting-state functional magnetic resonance imaging (fMRI) studies from task-independent modality.

Clusters	Regions	L/R	MNI coordinate			ALE score	*P*	Z	Voxels (mm^3^)	Jackknife sensitivity analysis
1	Superior temporal gyrus	R	64	−20	8	0.0173	5.19E-07	4.884	528	6/7

In the “Reported significant correlated regions” column, “L” refers to the left hemisphere, and “R” refers to the right hemisphere.

### 3.3 Structural MRI studies results from task-independent modality

**FIGURE 6:**
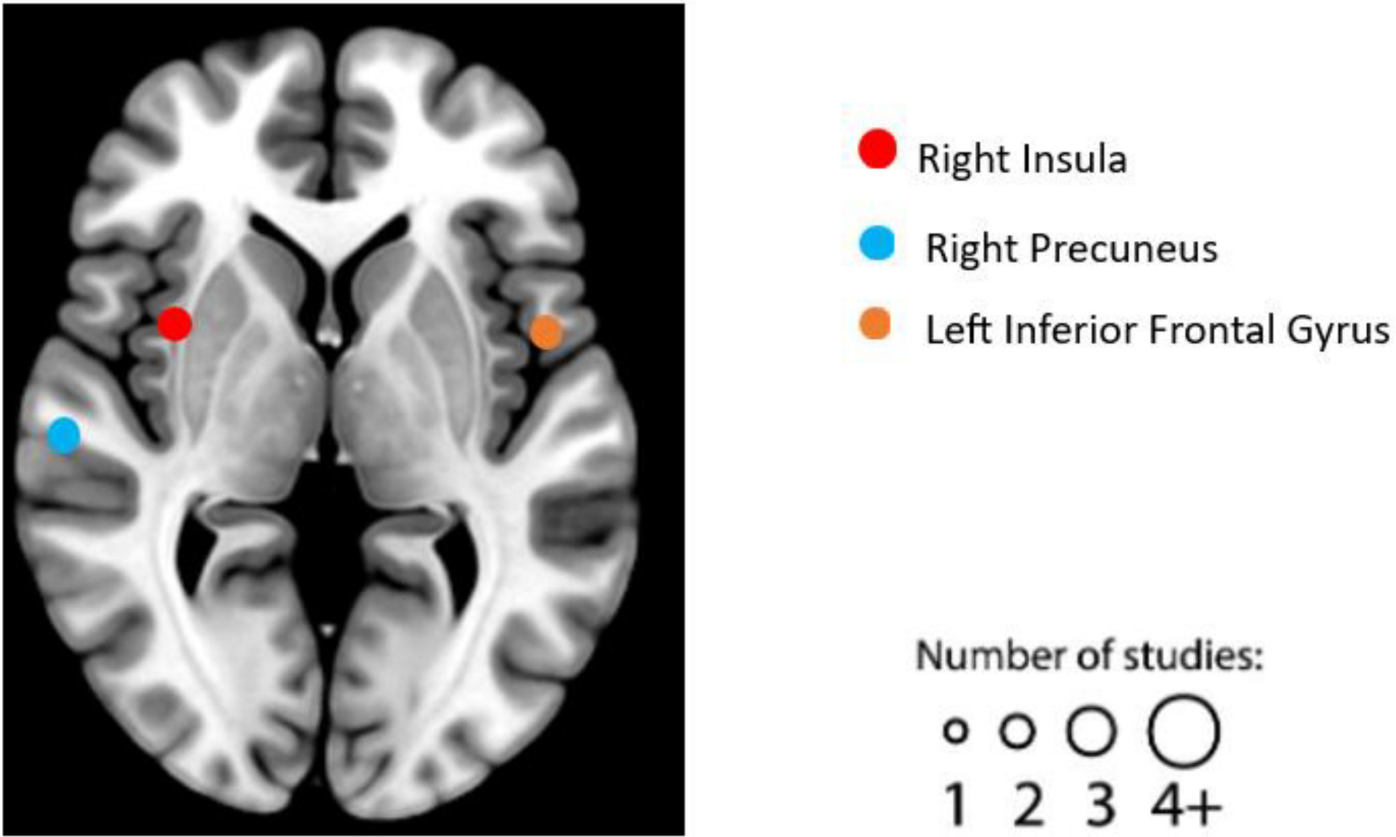
Graphical illustration of frequently reported structural magnetic resonance imaging (MRI) findings correlated with emotional well-being (EWB). Circles are sized according to the number of experiments reporting findings in the given location.

After literature screening and coding, seven individual experiments qualified in structural MRI studies. See Table 6 for details on the design, characteristics, and reported results of each experiment. We summarized the reported brain regions from the included experiments based on their frequency to identify patterns. Based on the characteristics of each included experiment, the most frequently reported brain regions correlated with EWB are the right insula (two individual experiments reported), right precuneus (two individual experiments reported), and left inferior frontal gyrus (two individual experiments reported) (see Figure 6). No significant cluster was found in this ALE meta-analysis of structural MRI studies.

**TABLE 6:** The design characteristics and results of task-independent modality [structural magnetic resonance imaging (MRI)] to emotional well-being (EWB).

References	Brain metrics	*N*	Age mean (SD)	Population	EWB measurement	Reported significant correlated regions
Matsunaga et al., 2016	Gray matter volume	106	21.40 (-)	University students	Subjective happiness scale	L_Rostral anterior cingulate cortex
Sato et al., 2015	Gray matter volume	51	22.50 (4.50)	Adults	Subjective happiness scale	R_Precuneus
Lewis et al., 2014	Gray matter volume	70	24.60 (3.76)	Healthy adults	42-item Ryff’s Scales Psychological Well-being	R_Insula
						R_Insula
						L_ Inferior Frontal Gyrus
						R_Medial Temporal Gyrus
						R_Insula
						R_Insula
						L_Insula
Rehbein et al., 2021	Gray matter volume	13	23.9 (3.4)	Pregnant women	Positive and Negative Affect Scale	L_Cuneus
Jung et al., 2022	Gray matter volume	70	36.6 (8.86)	Healthy individuals	World Health Organization Quality of Life Scale (WHOQOL-BREF)	R_Insula
Junca et al., 2023	Gray matter volume	25	46.0 (1.84)	Patients with Huntington’s disease	WHOQOL-BREF	L_Precuneus
						L_Inferior Frontal Gyrus
						R_Cerebellum
						L_Cerebellum
						L_Cerebellum
						R_Precuneus
						R_SupraMarginal Gyrus
						R_Precuneus
						R_Cerebellum
						R_Precuneus
						R_Cerebellum
Lu et al., 2023	Gray matter volume	126	22.65 (-)	University students	Psychological Wellbeing Scale	R_Inferior Frontal Gyrus

“-” denotes information not reported in the corresponding articles.

### 3.4 Shared and distinctive features between task-dependent and task-independent modalities of EWB

**FIGURE 7:**
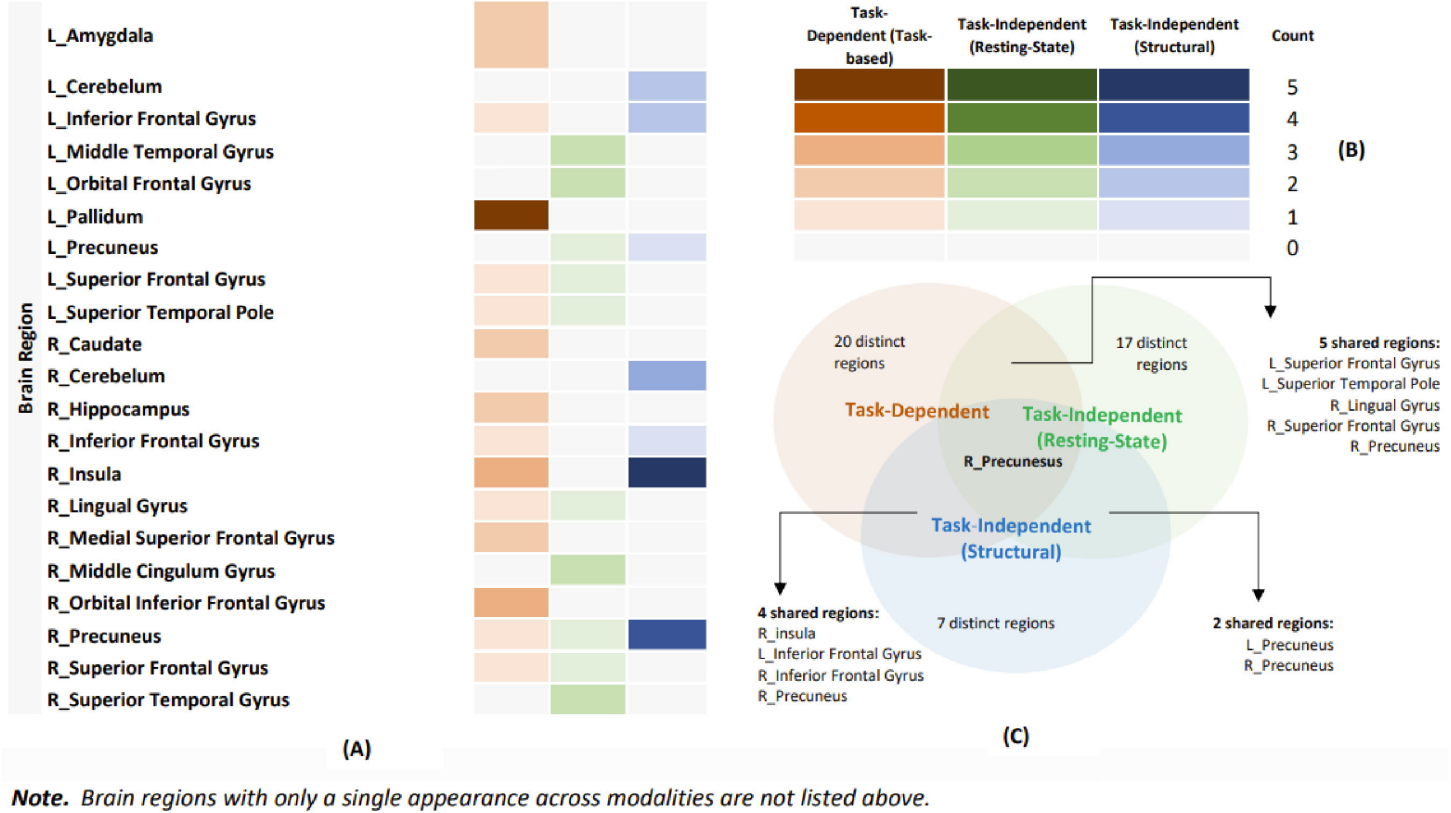
Illustration of shared and distinctive brain regions for each modality. **(A)** Illustration of the frequency with each brain region reported in each modality. **(B)** Orange represents task-based functional magnetic resonance imaging (fMRI), green represents resting-state fMRI, and blue represents structural magnetic resonance imaging (MRI). The color gradients indicate the frequency of reported brain regions. **(C)** A Venn diagram illustrating the overlap and distinctiveness of brain regions across various modalities.

When comparing task-dependent and task-independent modalities (task fMRI vs. resting-state fMRI; task fMRI vs. structural MRI), the ALE comparative analyses did not identify any shared or unique significant clusters. Additionally, the analysis comparing task fMRI and structural MRI could not be conducted due to an insufficient sample size. As previously mentioned, a descriptive approach was applied to all comparisons to highlight similarities and differences between modalities. As Figure 7 Venn diagram shows, the analysis identified several shared regions. The right precuneus was shared across task-dependent (task-based fMRI) and task-independent (resting-state fMRI and structural MRI) modalities. Between task-dependent (task-based fMRI) and task-independent (resting-state fMRI), shared regions included the left superior frontal gyrus, the left superior temporal pole, the right lingual gyrus, the right precuneus, and the right superior frontal gyrus. For task-dependent (task-based fMRI) and task-independent (structural MRI), the shared regions were the left inferior frontal gyrus, right precuneus, right inferior frontal gyrus, and right insula. Finally, comparing task-independent modalities (resting-state fMRI and structural MRI) revealed shared regions in the left and right precuneus. This descriptive analysis highlights the overlap and distinctions among different modalities, thereby contributing to a more nuanced understanding of the shared neural regions.

### 3.5 Neurosynth decoder- terms associated with the patterns of meta maps of EWB

**FIGURE 8:**
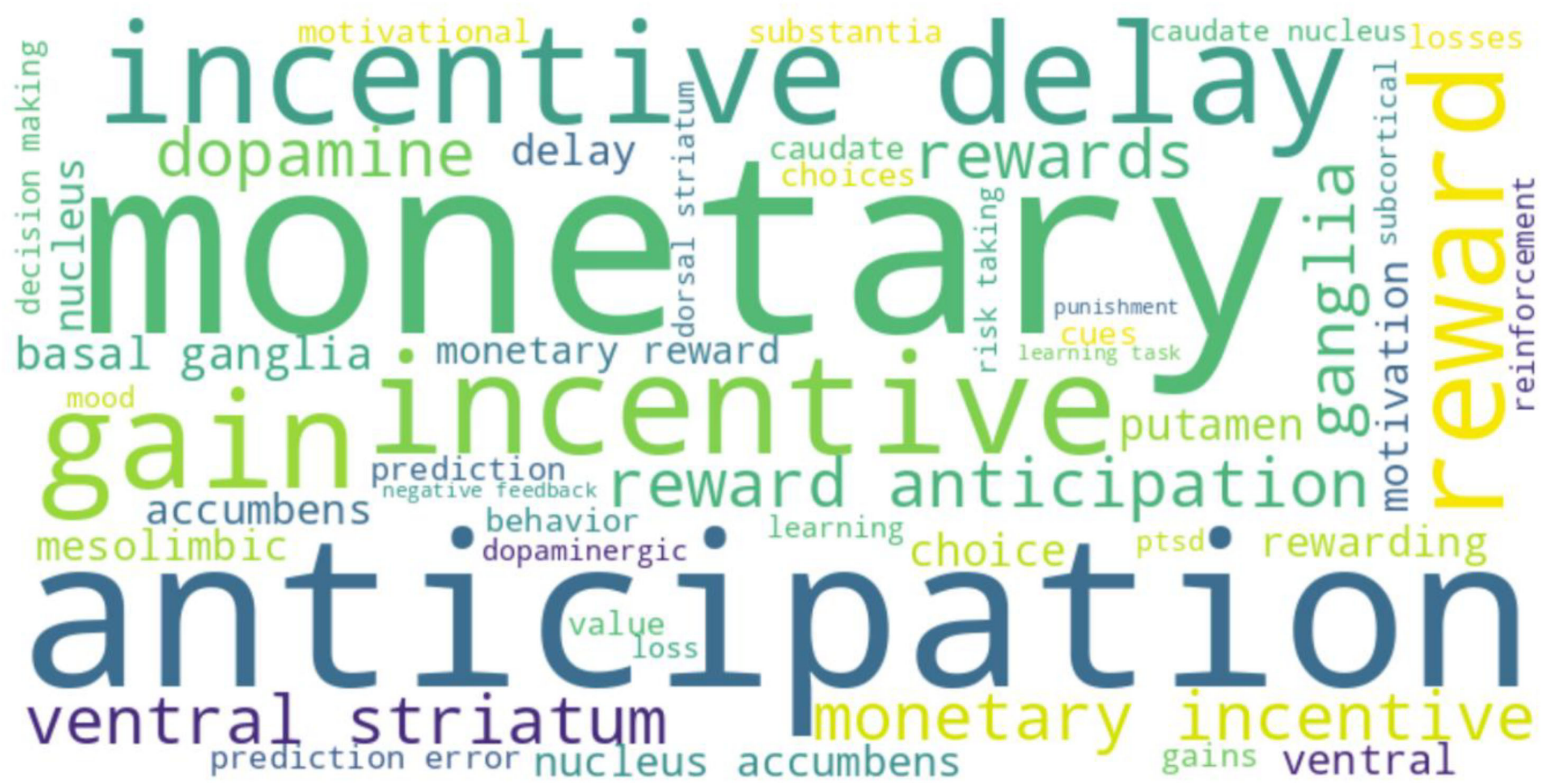
Decoding of the activation likelihood estimation (ALE) meta-maps from the task-dependent modality [task-based functional magnetic resonance imaging (fMRI) studies] using a Neurosynth decoder displayed on a word cloud. The font size represents the relative correlation strength; the different colors do not have any specific meaning.

**FIGURE 9:**
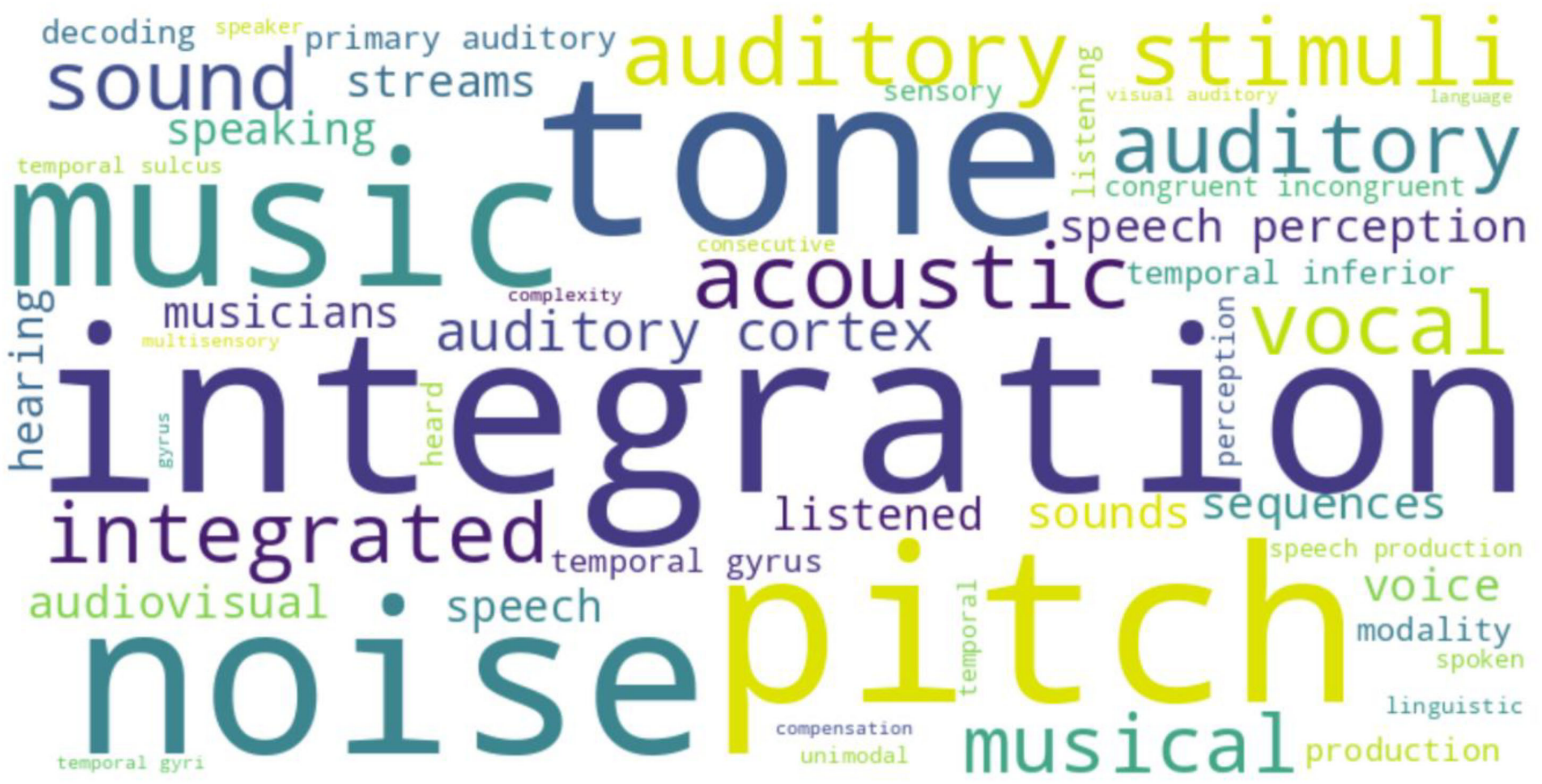
Decoding of the activation likelihood estimation (ALE) meta-maps from the task-independent modality [resting-state functional magnetic resonance imaging (fMRI) studies] using a Neurosynth decoder displayed on a word cloud. The font size represents the relative correlation strength; the different colors do not have any specific meaning.

Using the Neurosynth decoder, we provided the reverse inference analysis of ALE meta-maps from task-dependent (task-based fMRI) and task-independent (resting-state fMRI) studies. In the task-dependent studies, significant clusters were linked to terms such as monetary (strongest correlation: *r* = 0.21), incentive (*r* = 0.20), rewards (*r* = 0.17), rewards anticipation (*r* = 0.17), and dopamine (*r* = 0.17) (see Figure 8 for the word cloud). In the task-independent studies, significant clusters were associated with terms like integration (strongest correlation: *r* = 0.24), tone (*r* = 0.23), pitch (*r* = 0.22), and speech perception (*r* = 0.14) (see Figure 9 for the word cloud).

## 4 Discussion

This study provides the first preliminary systematic review and meta-analysis of the neural correlates of EWB using the 2023 NIH consensus definition. By separately examining task-dependent and task-independent MRI studies via a hypothesis-driven approach, we identified distinct patterns: the left pallidum in task-dependent modality, right STG and insula in the task-independent modality. Frontoparietal regions emerged across both modalities.

### 4.1 Task-dependent modality: the processes related to incentive and rewards regulation in EWB

The systematic review supplemented by meta-analysis of task-based fMRI studies revealed correlations between the activation of specific brain regions, the left pallidum, and EWB levels in fMRI tasks like emotion recognition or rewards processing. The pallidum regions are critical components of the brain’s rewards circuitry (Haber and Knutson, 2010). Interaction with other areas involved in dopamine release may affect an individual’s EWB levels by influencing the regulation of incentives and rewards. Dopamine release is critical for rewards processing and likely plays a significant role in EWB. Specifically, the left pallidum is involved in pleasure and positive emotional reinforcement, and rewards processing. (Šimić et al., 2021; Kringelbach and Berridge, 2009; Delgado et al., 2005) was found to be significantly correlated with EWB (Berridge and Kringelbach, 2015). Supporting this, the Neurosynth decoder of the task-based fMRI ALE maps identified correlated terms such as “incentive,” “rewards,” and “dopamine” (See Figure 7), which align with our hypothesis. Previous studies also suggested the pallidum’s involvement in EWB by regulating incentives and rewards (Lin et al., 2023). Research by Kringelbach and Berridge (2009), Berridge and Kringelbach (2015) identified conserved subcortical hedonic networks, including the ventral pallidum, where activation of specific neural “hotspots” enhances “liking” responses to pleasant stimuli. These subcortical circuits, involved in affective responses, have extensive interactions with prefrontal regions such as the anterior cingulate, orbitofrontal, and insular cortices.

In summary, our findings support the hypothesis that dopamine-related brain regions are crucial for task-dependent EWB processes, as they play a key role in regulating incentives and rewards. Individuals with higher/lower EWB levels may exhibit different dopamine-related brain responses when regulating incentives and rewards. The differences highlight the neural mechanisms underlying EWB and suggest that brain activity in dopamine-related regions during rewards-related processes could serve as a neural marker for individual differences in EWB.

### 4.2 Task-independent modality: the processes related to social cognition and interoceptive awareness in EWB

A systematic review and meta-analysis of resting-state fMRI revealed a correlation between EWB and spontaneous neural activity in the STG. Although we did not find a significant cluster in the meta-analysis of structural MRI studies, patterns in the insula offer insights into the underlying mechanism of EWB. Previous studies have found that STG, the brain region adjacent to the TPJ, is involved in emotion recognition, interpersonal interaction, understanding one’s own and others’ psychological states, and the perception of social information —all processes closely related to an individual’s EWB. This was validated in some content by the relevant term “tone” found in the Neurosynth decoder (See Figure 9); the tone of voice aids in interpreting emotions and intentions, which is crucial for understanding social cues and fostering effective communication during social interactions (Yager and Ehmann, 2006). The brain activation of the STG was related to social cognition or social perception (Blakemore, 2008). A genetic study (Song et al., 2019) also found that higher polygenic scores (PGSs) were significantly associated with increased brain structure in the right STG, which is involved in social cognition. In addition, the insula, consistently reported in the included structural MRI studies, is part of the salience network, integrates interoceptive sensations and emotional states, and may influence EWB through its role in social, affective, and cognitive processes (Paulus and Stein, 2006; Craig, 2009; Menon and Uddin, 2010). The insula is essential for monitoring internal states and evaluating external stimuli, which are both critical for maintaining EWB. Its role in social interactions and emotional experiences links physical states to emotional and social cognition, potentially directly influencing an individual’s level of EWB.

In conclusion, our study of the neural correlates of EWB in the task-independent modality highlights the central roles of the STG and insula, which are key to the processes of social cognition and interoceptive awareness. These regions likely contribute to EWB by shaping social understanding and self-awareness, both of which are essential for maintaining mental states and self-evaluative processes that influence overall EWB.

### 4.3 Shared and distinctive neural patterns suggest the complexity of EWB

When comparing task-dependent and task-independent modalities, although no shared or distinctive significant clusters were identified in the ALE comparative analysis, possibly due to insufficient power to detect the patterns or due to Type II error arising from the limitation in task-dependent studies, and the use of thresholded maps. However, in the descriptive comparison between different modalities, during the comparison between task-dependent (task-based fMRI) and task-independent (resting-state fMRI and structural MRI) modalities, we found the common shared region (right precuneus) in the frontoparietal area, which is responsible for higher-order cognitive functions and maintaining mental health (Cole et al., 2014), has been found to be a key hub in the default mode network supporting EWB (Bian et al., 2024). Additionally, task-based fMRI and resting-state fMRI, as well as task-based fMRI and structural MRI, reveal overlapping regions in the frontoparietal area, including the superior and inferior frontal gyri. This suggests that cognitive control processes may play an overarching role in supporting EWB, a finding also supported by previous studies. Lewis et al. (2017) found that higher cognitive control abilities correlated with greater scores in purpose in life, one of the key domains of EWB (Park et al., 2023). Furthermore, longitudinal studies demonstrate that better processing speed at baseline is associated with higher life satisfaction (one of the key domains of EWB) in the future (Enkvist et al., 2013).

Regarding distinctive neural patterns, no statistically convincing results were found in the ALE comparative analysis. However, descriptive comparisons indicate that the proportion of distinctive brain regions is higher in task-dependent than task-independent studies (see Figure 7). While this evidence is based on descriptive comparisons, it may suggest that EWB-related neural processes in task-independent modalities are less specific, likely due to resting-state fMRI and structural MRI capturing more widespread intrinsic activity and structure (Smith et al., 2013), resulting in more diffuse associations with EWB. For instance, during resting-state fMRI, participants are not engaged in specific tasks, which results in broader brain activity patterns that are harder to attribute directly to EWB. Additionally, the distinction between “state” and “trait” may help explain these differences. The task-dependent modality may better capture “state” variations of EWB (e.g., positive affect), reflecting how someone feels at a specific moment, which is more tied to distinctive brain regions. Task-independent modalities, such as resting-state fMRI and structural MRI, may better reflect the “trait” stable aspects of EWB (e.g., quality of life), which are less tied to specific brain regions. The evidence for supporting the distinction between “state” and “trait” of EWB has been found in previous studies; a scoping review found that “trait” well-being (i.e., the propensity to live according to one’s true nature) is associated with various brain regions and not consistently tied to any specific area (King, 2019), possibly due to only focusing on task-independent modalities that capture “trait” aspects of EWB, which are less connected to specific brain regions. This interpretation suggests that task-dependent modalities may better capture “state” variations in EWB, while task-independent modalities may better reflect the “trait,” stable aspects of EWB. However, the definitions of “state” and “trait” in the context of EWB remain unclear. Therefore, future empirical research is needed to elucidate the complex neural mechanisms underlying EWB.

### 4.4 Limitations

There are several limitations to the current study that should be considered. Firstly, this current study primarily focuses on fMRI and MRI studies. Electroencephalography (EEG) and event-related potentials (ERPs) are not included in meta-analyses, and these modalities may be better suited for examining the temporal aspects of EWB. Functional near-infrared spectroscopy (fNIRS) is another promising modality which we did not include in this study, although its portability and suitability for developmental and naturalistic settings make it an important avenue for future research. Future research should incorporate other imaging methods, beyond fMRI and MRI, to enable further exploration of the neural mechanisms underlying EWB. Secondly, our study aimed to include a broad range of populations to explore EWB across different demographics and conditions as in prior systematic reviews. However, this diversity may have introduced heterogeneity, increasing the risk of false positives or negatives. Future research should recognize that the neural mechanisms of EWB may differ across groups.

In our analysis, Egger’s test revealed publication bias (Egger et al., 1997), a common issue in meta-analyses, suggesting that our findings should be interpreted with caution. However, this also reflects the limitations in the field of EWB research. According to the quality assessment of included literature, 16% reported only the names of the brain regions significantly associated with EWB without reporting the specific coordinates; 23% of the studies did not report the cluster sizes of the significant brain regions they found; and none of the studies provided the un-thresholded map, including effect size images (Poldrack et al., 2008). The lack of standardized reporting in these previous studies limits their reproducibility. This publication bias highlights the need to adopt standardized protocols to reduce bias and enhance the robustness of future EWB research. Some resting-state fMRI individual experiments included in this study originated from the same research group (Kong et al., 2015a, 2015b, 2015c, 2016, 2018). Due to the lack of detailed cohort reporting in their papers, a potential sample overlap cannot be ruled out; however, our findings should be interpreted with caution. Future studies should enhance cohort transparency to allow a clearer assessment of dataset independence.

In addition to identifying brain regions associated with EWB, our study initially aimed to conduct separate meta-analyses to distinguish the distinct contributions of positive and negative correlations with EWB scores. However, due to the limited sample size, we combined studies reporting both positive and negative correlations in the main analysis, ensuring a careful interpretation of the findings and a transparent discussion. Additionally, as only task-fMRI studies reported positive correlations, which provided sufficient statistical power for the separate analysis, we conducted a supplementary analysis for this subset, identifying significant clusters in the left pallidum (see Supplementary Figure 2 and Supplementary Table 5). This finding reinforces the critical role of dopamine-related regions supporting EWB in the task-dependent modality. However, the methodology of combining studies regardless of correlation direction could also be a limitation, as it may mask specific effects or inflate results. Nevertheless, due to practical considerations, we had to combine them. Future meta-analyses, when more literature is available, should take directionality into account.

Due to the absence of task paradigms that directly measure EWB so far, the analysis in the current study relies on brain regions that correlate between self-report of EWB and existing task paradigms in the task-dependent modality, such as emotion recognition (44%), rewards/incentives (19%), and other tasks (37%). This may have introduced bias into the findings. For example, in rewards/incentive paradigms, collapsing responses to both positive and negative stimuli can reduce analytical power and complicate interpretation. An increased rewards response to negative stimuli might suggest a person finds negative experiences reinforcing, which could harm their EWB. In contrast, an increased response to positive stimuli likely indicates a beneficial reaction that enhances EWB, so caution is needed when interpreting these findings. Furthermore, in our systematic review and meta-analysis of task-dependent modality, all the task-dependent studies included began with whole-brain analysis before narrowing their focus to clusters that showed significant activation, and then used these clusters to correlate with EWB scores. Although this approach deviates from the ideal of identifying un-thresholded whole-brain maps to correlate with EWB, we believe it can still reveal important information. No prior systematic reviews have synthesized neural correlates from a task-dependent perspective, so our study fills this gap by conducting an in-depth analysis. Furthermore, this comprehensive review will enhance understanding and inform future research. However, the risk of a Type II error, such as important regions that are not significantly activated at the group level by the specific tasks used, may still correlate with the EWB score across individuals. This limitation, combined with our limited power, means that our findings should be interpreted as a starting point for EWB research rather than an exhaustive list of regions involved in EWB. Additionally, developing EWB-specific paradigms could also provide deeper insights and help reduce these limitations.

In our analysis of EWB measures used in the included studies (see Supplementary Table 2), the Positive and Negative Affect Scale (PANAS) was the most used, followed by the Life Satisfaction Scale and the Subjective Happiness Scale. This suggests that research on the neural mechanisms of EWB has primarily focused on certain domains of EWB, such as positive affect and life satisfaction. However, the sense of meaning domain has received comparatively less attention, highlighting a gap in the current research landscape. Most studies have focused on only one domain of EWB, which may contribute to inconsistencies across findings that examine different domains. Future research should recognize EWB as a multifaceted construct and study its various domains to gain a more comprehensive understanding. Adopting a holistic approach that addresses and delineates different components of EWB, rather than treating it as a single, broad construct, will provide deeper insights into its neural mechanisms.

### 4.5 Implications

**FIGURE 10:**
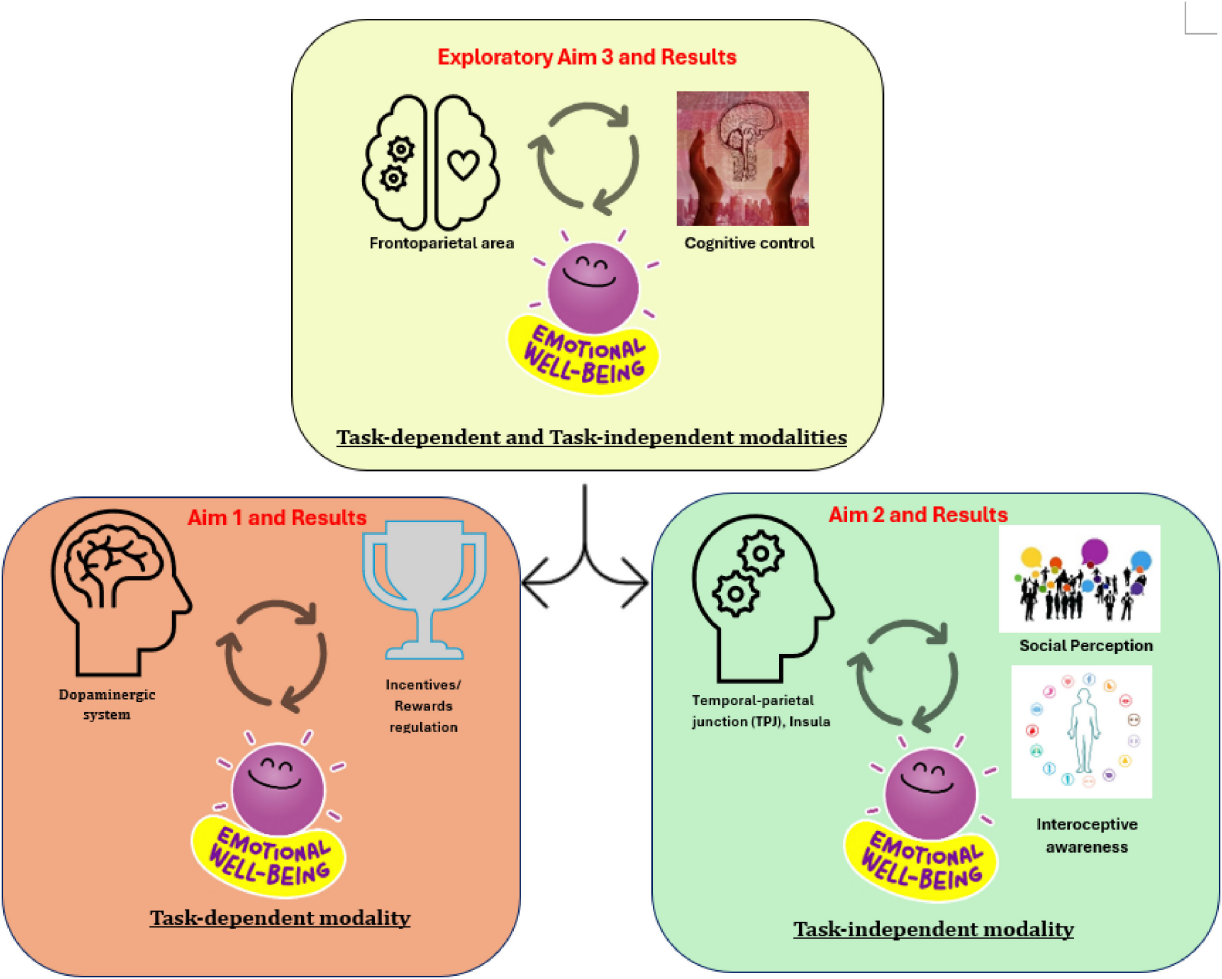
The proposed framework illustrates possible neural mechanisms underlying EWB (emotional well-being) across different magnetic resonance imaging (MRI) modalities based on key brain regions identified in this study.

This study advances understanding of the neural correlates of EWB by examining both task-dependent and task-independent MRI modalities. The findings provide modality-specific neural markers that address limitations of subjective measures and may guide practical applications. Clinically, therapists could develop treatments focusing on regions like the STG to enhance social cognition and improve EWB. In educational and workplace contexts, tasks engaging rewards-related and cognitive control processes could support EWB. Building on Figure 10, future studies could develop tasks targeting processes such as incentive and rewards regulation, social cognition, interoceptive awareness, and cognitive control. This would move beyond self-report questionnaires, which are vulnerable to biases like social desirability and self-perception errors (King, 2019). Although current evidence is limited by sample size, larger-scale studies are needed to validate and extend this neural framework, reinforcing both its theoretical and translational value.

## 5 Conclusion

In summary, this study presents the first preliminary systematic review and meta-analysis of the neural correlates of EWB using the 2023 NIH consensus definition. Task-dependent analyses identified the pallidum, underscoring its role in incentive and rewards regulation, while task-independent analyses implicated the STG and insula in social cognition and interoceptive awareness. Across modalities, the frontoparietal region emerged as a shared substrate supporting cognitive control. Together, these findings provide a preliminary integrative framework for the neural basis of EWB and highlight directions for future large-scale studies to refine paradigms and guide interventions that promote EWB.

- The orange module (Aim 1, task-dependent modality): Illustrates the pallidum’s role in supporting EWB via rewards regulation (see Figures 2, 3 and Table 3).
- The green module (Aim 2, task-independent modality) Shows the roles of the insula and STG (closely located to the TPJ) in social cognition and interoceptive awareness, contributing to EWB (see Figures 4–6 and Table 5).
- The yellow module (Exploratory Aim 3, both modalities) shows shared brain regions in the frontoparietal areas, which may support EWB through cognitive control processes (see Figure 7).

## Data Availability

The data analyzed in this study is subject to the following licenses/restrictions: this study is a systematic review and meta-analysis. All data analyzed in this study were obtained from previously published articles, as cited, and are available in their respective sources. No new data were generated or collected for this study. Requests to access these datasets should be directed to jie.luo@uconn.edu.

## Footnotes

1
https://m3ewb.research.uconn.edu/ewb-subjective-measures-repository/

2
https://bioimagesuiteweb.github.io/webapp/mni2tal.html

3
https://osf.io/ymtb8/

4
http://brainmap.org/ale/

5
https://neurosynth.org

6
https://neurovault.org/
