# Supplementary material for: Human neural correlates of emotional well-being (EWB): a preliminary systematic review and meta-analysis of MRI studies based on a recent consensus definition
Source: Front Hum Neurosci. 2025 Nov 10;19:1669164. doi: 10.3389/fnhum.2025.1669164 (PMC12640920; doi:10.3389/fnhum.2025.1669164)
Supplement: Supplementary file 1 [file Data_Sheet_1.docx]

## **Supplementary Materials**

| **Table S1.**  *Specific quality assessment questions* | |
| --- | --- |
| **Clear research hypotheses/Rational/Justification/Design** | Did the study have clear Hypotheses/Rational/Justification/Design for the relationship between emotional Well-being (EWB) and the brain? |
|  | What are the clear Hypotheses/Rational/Justification/Design for the relationship between emotional Well-being (EWB) and the brain? |
|  | What score would you give for the quality of this study based on research Hypotheses/Rational/Justification/Design? |
| **Power analysis for its sample size** | Did authors explicitly report possible power given its sample size? |
| **Standardized fMRI data preprocessing analysis procedure** | How did this study do the nuisance regressors during the data preprocessing? |
|  | How did this study do the motion correction? |
|  | Did they report motion parameters? |
|  | What are the motion parameters they reported? |
|  | Did they control for motion parameters in group level/between subject analyses? |
|  | How did this study control for motion parameters in group level/between subject analyses)? |
|  | Did this study do the temporal filtering (or called temporal band pass filtering) during the data processing? |
|  | Did this study do smoothing in the data analysis? |
|  | What is the full-width at half-maximum (FWHM) they used for the smoothing? |
|  | What score would you give for the quality of this study based on the fMRI data preprocessing analysis procedure? |
| **Reported details of the results of this study** | Did the brain regions significantly associated with EWB in this study provide peak coordinates, peak values, cluster sizes or effect sizes? |
|  | Did this study do Multiple Comparison Corrections when performing the data analysis? |
|  | Which method did they use for the Multiple Comparison Corrections? |
|  | Did they report a threshold of significance for reporting significant brain regions related to EWB? |
|  | What is the voxel threshold they reported? |
|  | What is the cluster threshold they reported? |
|  | What score would you give for the quality of this study based on the reported details of the results of this study? |
| **Data available** | In this paper, was the data available? |
|  | In this paper, was the data analysis code available? |
|  | What score would you give for the quality of this study based on the data available? |

| **Table S2**.  *EWB measurement and domains: frequency in previous EWB neuroimaging studies* | | | | | |
| --- | --- | --- | --- | --- | --- |
| **Task-dependent modality** | | | **Task-independent modality** | | |
| **EWB Measurement** | **EWB Domain Measured** | **Frequency** | **EWB Measurement** | **EWB Domain Measured** | **Frequency** |
| Positive and Negative Affect Scale | Positive Affect, Life Satisfaction | 9 | Positive and Negative Affect Scale | Positive Affect, Life Satisfaction | 4 |
| Satisfaction with Life Scale | Life Satisfaction | 3 | Satisfaction with Life Scale | Life Satisfaction | 3 |
| Subjective Happiness Scale | Life Satisfaction; Positive Affect | 3 | Subjective Happiness Scale | Life Satisfaction; Positive Affect | 3 |
| WHO Quality of Life Brief Scale | Quality of Life, Life Satisfaction, Sense of Meaning | 1 | Psychological Well-being | Goal Pursuit; Life Satisfaction; Positive Affect; Sense of Meaning | 3 |
| Psychological Well-being | Goal Pursuit; Life Satisfaction; Positive Affect; Sense of Meaning | 2 | Ryffs Scales Psychological Well-being | Goal Pursuit; Life Satisfaction; Positive Affect; Sense of Meaning | 2 |
|  |  |  | WHOQOL-BREF | Life Satisfaction; Quality of Life; Sense of Meaning | 2 |

| **Table S3.**  *Results of the jackknife sensitivity analyses of the meta-analysis in Task-based fMRI studies* | |
| --- | --- |
| Study | Left Pallidum |
| Heller (2013) | Yes |
| Shi (2016) | Yes |
| Zhang (2014) | Yes |
| vanReekum (2007) | Yes |
| Sanchez (2015) | Yes |
| Park (2017) | No |
| Park (2017) | Yes |
| Morelli (2018) | Yes |
| Memarian (2017) | Yes |
| Matsunaga (2016) | Yes |
| Martin-Soelch (2021) | Yes |
| Martin-Soelch (2021) | Yes |
| Killgore (2007) | Yes |
| Fonzo (2017) | Yes |
| Mathiak (2013) | Yes |
| Murray (2023) | Yes |

| **Table S4.**  *Results of the jackknife sensitivity analyses of the meta-analysis in Resting-state fMRI studies* | |
| --- | --- |
| Study | Right Superior temporal gyrus |
| Kong (2015A) | Yes |
| Sato (2019) | Yes |
| Kong (2018) | Yes |
| Kong (2016) | Yes |
| Kong (2015B) | No |
| Kong (2015C) | Yes |
| Li (2022) | Yes |


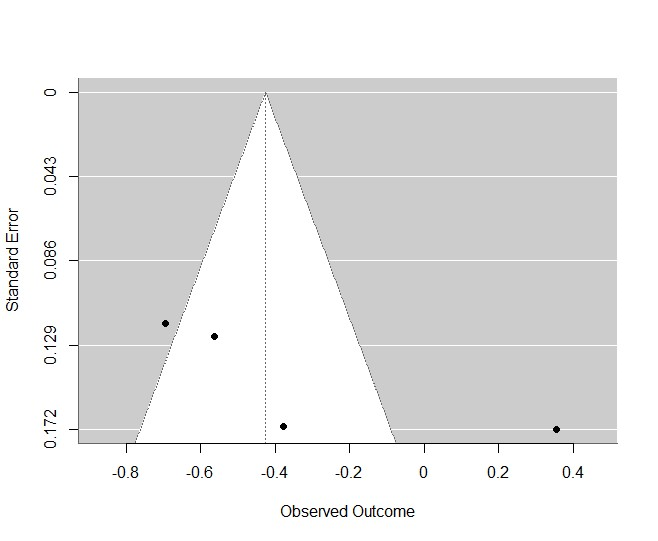


**Figure S1.** Results of funnel plot analyses to test for publication bias. A significance level of *p* < 0.05 for Egger's test indicated the presence of significant publication bias in the current study. For significant clusters found in task-dependent studies, Egger's tests were significant (*p* < 0.05) for the left pallidum (*p* < 0.0001), which means publication bias existed. In the task-independent studies, there are not enough sample sizes to perform Egger's tests.

**ALE analysis of task-fMRI Studies reporting only positive correlations**

To identify brain regions associated with EWB, it is essential to recognize the distinct contributions of positive and negative correlations with self-reported EWB scores. Analyzing these separately can uncover nuanced psychological and neurobiological processes, as separate handling of correlations provides clearer insights into brain correlates. Although our study aimed to conduct separate analyses for these correlations, the limited sample size posed a significant challenge. To address this limitation, we combined studies reporting both positive and negative correlations in the main analysis, ensuring careful interpretation of the findings and a transparent discussion in the results section. Furthermore, we performed supplementary analyses on studies reporting exclusively positive or negative correlations with sufficient statistical power, as detailed below.

| **Table S5.**  *EWB meta-maps in task-based fMRI studies reported positive correlation from task-dependent modality* | | | | | | | | | | |
| --- | --- | --- | --- | --- | --- | --- | --- | --- | --- | --- |
| **Clusters** | **Regions** | **L/R** | **MNI coordinate** | | | **ALE Score** | **P** | **Z** | **Voxels (mm3)** | **Jackknife sensitivity analysis** |
| 1 | Pallidum | L | -16 | 8 | 0 | 0.0177 | 3.01E-08 | 5.4185 | 1032 | 12/13 |
|  | Amygdala | L | -24 | -4 | -10 | 0.009 | 7.06E-05 | 3.8062 |  |  |
|  | Pallidum | L | -22 | 2 | -6 | 0.009 | 7.10E-05 | 3.805 |  |  |
| ***Note.*** In the resting-state fMRI studies, there is not enough sample size to conduct the separate analysis based on the directions of the correlations. Since the sample size requirement of the ALE meta-analysis, only the task-fMRI studies with positive correlations can be analyzed. | | | | | | | | | | |


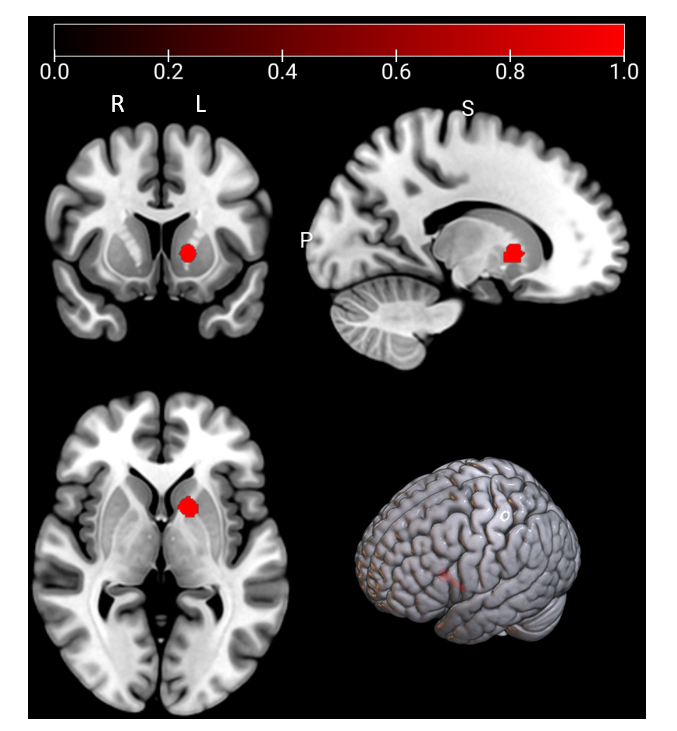


**Figure S2.** Emotional well-being (EWB) meta-maps (marked with red color) of task-based fMRI studies reported positive correlations (task-dependent modality). “L” refers to the left hemisphere, and “R” refers to the right hemisphere. Same for the following figures.
